# Adipocyte phosphatidylinositol biosynthesis via the Lands cycle protects against insulin resistance

**DOI:** 10.1016/j.jlr.2023.100383

**Published:** 2023-04-29

**Authors:** Karin E. Bornfeldt

**Affiliations:** Department of Medicine, Division of Metabolism, Endocrinology and Nutrition, UW Medicine Diabetes Institute and Department of Laboratory Medicine and Pathology, University of Washington, Seattle, WA, USA

The Lands cycle, elucidated by Dr William (Bill) Lands, describes the recycling of phospholipids by addition of a fatty acyl chain to a lysophospholipid by lipid acyltransferases and cleavage of a fatty acyl chain to generate a lysophospholipid by phospholipases (1). Dr Lands published a series of elegant biochemical studies in the late 1950s to mid-1960s (e.g., (2)), describing these processes (Fig. 1). The Lands cycle allows a cell to remodel the acyl chain composition of its membrane phospholipids, and thereby its physical and functional properties (1). Such remodeling is critical, as recent research demonstrates a role for the Lands cycle in metabolic disease.

**Fig. 1:**
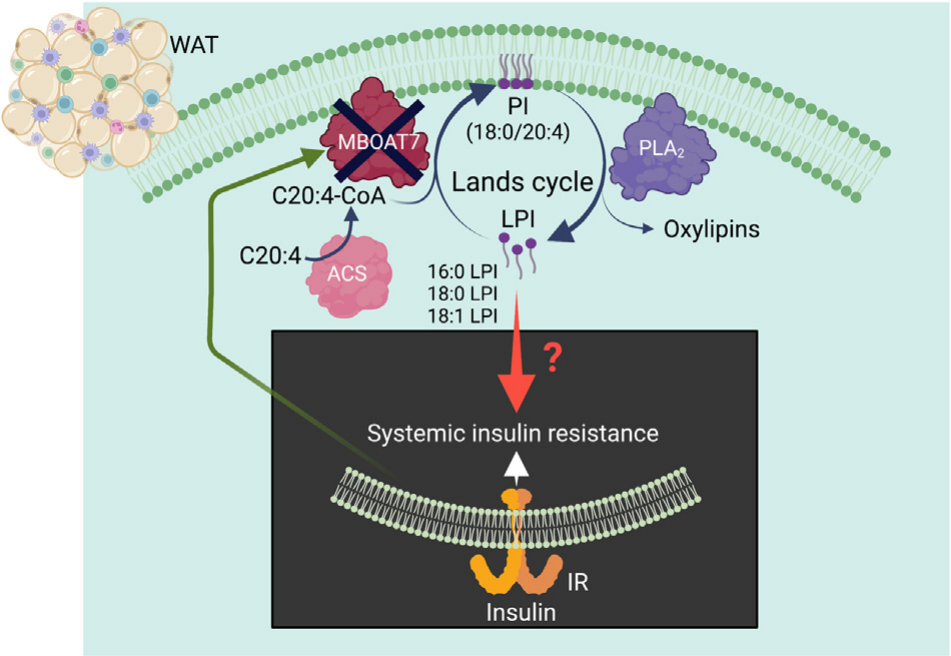
How does phosphatidylinositol biosynthesis via the Lands cycle in white adipose tissue prevent systemic insulin resistance? The findings by Massey *et al.* (3) show that deletion of MBOAT7 expression selectively in white adipose tissue (WAT) results in systemic insulin resistance in fat-fed mice, suggesting that MBOAT7-mediated PI biosynthesis through the Lands cycle in WAT is required for systemic insulin sensitivity. The mechanism whereby the WAT MBOAT7-mediated Lands cycle modulates systemic insulin resistance is still largely a “black box” and needs further investigation. While data provided by Massey and colleagues support the proposal that the insulin resistance is due to autocrine or paracrine effects of 16:0-, 18:0-, or 18:1-lysophosphatidylinositol (LPI) accumulated in WAT, other interpretations are possible. Interestingly, MBOAT7 expression is downregulated by insulin (4), potentially worsening insulin resistance. ACS, acyl-CoA synthetase; IR, insulin receptor; PLA_2_, phospholipase A_2_. Created with BioRender.com.

One of the mammalian lysophospholipid acyltransferases is termed MBOAT7 (membrane bound O-acyltransferase domain containing 7). An updated nomenclature has recently been proposed, which renames MBOAT7 as LPLAT11 (lysophospholipid acyltransferase 11) (5). MBOAT7 has attracted increased interest in the area of metabolic disease because genome-wide association studies have identified a susceptibility locus (rs641738) within a linkage-disequilibrium block that contains the *MBOAT7* gene that associates with liver disease, including nonalcoholic fatty liver disease (6).

MBOAT7 is highly selective in esterifying lysophosphatidylinositol (LPI) to arachidonoyl-CoA (C20:4-CoA), generating phosphatidylinositol (PI[18:0/20:4]), as shown in Fig. 1. Consistent with the genome-wide association study, silencing MBOAT7 by an antisense oligonucleotide, which resulted in lower MBOAT7 expression primarily in liver, adipose tissue, and cells within the reticuloendothelial system, caused a nonalcoholic fatty liver disease phenotype in fat-fed mice (7). Although there were no detected differences in body weights or circulating lipoproteins, MBOAT7 silencing also resulted in insulin resistance, characterized by glucose intolerance, elevated plasma insulin and C-peptide levels, and a reduced ability of insulin to induce insulin receptor β-subunit phosphorylation and downstream Akt phosphorylation in hepatocytes. The dampened insulin action in liver was not observed in adipose tissue, despite an almost similar silencing of MBOAT7 expression in white adipose tissue (WAT) of these mice (7). The proposed mechanism was suggested to be due to increased hepatic lipid droplet expansion, but it remained unclear how this mechanism would inhibit insulin’s action.

However, when MBOAT7 deletion was subsequently introduced specifically in hepatocytes, the fatty liver phenotype observed in the MBOAT7 antisense oligonucleotide studies above was replicated, but not the insulin resistance (8). One possibility, therefore, was that suppression of MBOAT7 expression in adipose tissue could explain the insulin resistance observed by Helsley and colleagues (7).

It is in light of these findings that the study by Massey and colleagues (3) finds its important context. Having previously demonstrated that hepatic expression of MBOAT7 is suppressed in obese humans and rodents (7), that *Mboat7* expression in WAT is negatively correlated both with fat pad weight and percent body fat in mice, and that *Mboat7* expression in WAT negatively associates with indices of insulin sensitivity, the group confirmed these findings by demonstrating that *Mboat7* expression in WAT is negatively correlated with WAT mass in mice fed a high-fat high-sucrose diet, although sex differences were evident in the fat depots affected.

To dissect the effects of MBOAT7 in liver and adipose tissue, the group generated adipocyte-targeted and hepatocyte-targeted MBOAT7-deficient mice using *Mboat7* floxed mice crossed with adiponectin-Cre and albumin-Cre mice, respectively. Analysis of liver pathology and insulin resistance in these mice revealed that liver-targeted deletion of MBOAT7 resulted in a fatty liver phenotype but not insulin resistance, consistent with previous studies (8). Conversely, although fasting plasma insulin levels were elevated in fat-fed mice lacking MBOAT7 in hepatocytes, deletion of MBOAT7 in adipocytes caused hyperinsulinemia and reduced insulin sensitivity, with only minor effects on the liver fat. Thus, the insulin resistance phenotype appears to be largely selective to loss of MBOAT7 in adipocytes. Interestingly, MBOAT7 is downregulated by acute injection of insulin in both liver and adipose tissue (4), suggesting the possibility that hyperinsulinemia might worsen systemic insulin resistance through downregulation of MBOAT7.

The study by Massey and colleagues (3) raises many intriguing questions, including questions on the mechanism whereby adipocyte MBOAT7-deficiency worsens insulin resistance in fat-fed mice and whether the insulin resistance phenotype in adipocyte-targeted MBOAT7-deficient mice is due to adipocyte insulin resistance. To start to address the latter question, the authors performed hyperinsulinemic-euglycemic clamps. These studies indicated that the insulin resistance in adipocyte-targeted MBOAT7-deficient mice likely is due to autocrine or paracrine mechanisms leading to insulin resistance in the adipose tissue rather than in the liver or other insulin target tissues.

Massey further showed that MBOAT7 deficiency results in a marked reduction in PI species containing arachidonoyl-acyl chains, primarily PI(18:0/20:4), and increased levels of 18:0-LPI and 18:1-LPI in WAT (Fig. 1). But how would these changes lead to insulin resistance? Is it possible that reduced PI biosynthesis through the Lands cycle could lead to reduced insulin signaling through phosphatidylinositol phosphate kinases? Could altered levels of protein lipid anchors through glycosylphosphatidylinositol be an explanation? Answers to these questions await further research, as pointed out by the authors. In the meantime, several potential mechanisms mediated by changes in specific lipid species in WAT were ruled out by Massey *et al.* (3). A comprehensive lipidomics analysis of WAT showed that other than a few alterations in phospholipids containing polyunsaturated acyl-chains, changes of free fatty acids, non-LPI lysophospholipids, phosphatidylcholines, sphingomyelins, ceramides, triacylglycerols, diacylglycerols, or oxylipins were unlikely to explain the increased insulin resistance. Moreover, plasma nonesterified fatty acid levels did not appear to be increased in mice with adipocyte-selective MBOAT7 deficiency, suggesting that increased adipocyte lipolysis did not explain systemic insulin resistance.

There were no significant differences in liver or plasma levels of PI or LPI lipid species in mice lacking MBOAT7 in adipocytes, pointing to a local adipose tissue mechanism. To test if LPI indeed affects insulin signaling in WAT, mice with and without adipose MBOAT7 deficiency were injected with 18:1-LPI and the gonadal WAT global phosphoproteome was analyzed. Pathway enrichment analysis showed increased insulin signaling and insulin resistance signatures in the LPI-injected MBOAT7-deficient mice. Although direct measurements of insulin-induced Akt and PI3K activities in WAT were not done in the current study, these results support the authors’ conclusion that accumulation of LPI locally in adipose tissue alters insulin signaling in an MBOAT7-dependent manner.

Still, the mechanism whereby this occurs largely remains a black box mystery (Fig. 1). In addition to the mechanisms discussed above, a possibility may be that the increased adipose tissue inflammation (3), including a Cd11c^-^/Cd206^+^ cell population (an adipose tissue macrophage population expressing scavenger receptors in humans; (9)) and a reciprocal decrease in Cd11c^+^/Cd206^+^ double-positive cells (an antigen-presenting lipid-loaded adipose tissue macrophage population in humans; (9)), and higher numbers of T and B cells could contribute to insulin resistance. A causal relationship between adipose tissue macrophages and systemic insulin resistance has been shown by others (10). Such effects might potentially be mediated by the LPI receptor GPR55, which has been suggested to promote inflammatory activation of macrophages in response to LPI (11), through a paracrine effect of LPI released from adipocytes locally in the adipose tissue depot.

Undoubtedly, the study by Massey and colleagues will inspire more research into the mechanisms whereby MBOAT7 and PI biosynthesis through the Lands cycle protect against liver disease, adipose tissue inflammation, insulin resistance, and potentially diabetes.

## Conflict of interest

K.E.B. serves on the scientific advisory board of Esperion Therapeutics, Inc.
